# Personality Traits and Coping Strategies as Psychological Factors Associated with Health-Related Quality of Life in Highly Sensitive Persons

**DOI:** 10.3390/ijerph20095644

**Published:** 2023-04-26

**Authors:** Manuela Pérez-Chacón, Mercedes Borda-Mas, Antonio Chacón, María Luisa Avargues-Navarro

**Affiliations:** 1 Spanish Association of Highly Sensitive Psychologists and Professionals, PAS España, 28080 Madrid, Spain; 2 Department of Personality, Assessment, and Psychological Treatment, University of Seville, 41018 Seville, Spain

**Keywords:** sensory-processing sensitivity, neuroticism, extraversion, conscientiousness, mental health, vitality, emotional role functioning

## Abstract

Sensory-Processing Sensitivity (SPS) is the reactivity to different stimuli that occurs in some people with sufficient intensity to cause interference in daily life. There are not many previous studies that determine the influence of adaptive and maladaptive coping strategies on health-related quality of life through indicators of mental (anxiety and depression) and physical (vitality) health and functioning in their lives in different contexts (emotional role functioning). In this sense, contexts that promote the use of successful stress-coping strategies are related to the presence of positive mental health outcomes. This study focuses on the analysis of indicators of health-related quality of life in people with SPS in relation to certain personality traits and coping strategies. Participants (N = 10,525) completed HSPS-S, NEO-FFI, CSI, and SF-36. Differences were observed between men and women. Differences indicated that women had higher SPS scores compared to men and poorer health-related quality of life. The results showed significant relationships with the three indicators of health-related quality of life. Finally, it is confirmed that neuroticism and the use of maladaptive coping strategies act as risk factors, whereas extraversion, conscientiousness, and adaptive coping strategies act as protective factors. These findings highlight the need to develop prevention programs for highly sensitive persons.

## 1. Introduction

Sensory-Processing Sensitivity (SPS) is the reactivity produced in some people to different stimuli with sufficient intensity to produce disturbance in everyday life [1]. It is conceptualized as a personality trait characterized by deep information processing, increased awareness of environmental subtleties, increased emotionality and empathy, and ease of central nervous system overstimulation [2] associated with other personality traits (neuroticism and extraversion) [3].

In people with SPS, this biologically based trait [4] explains the excessive emotional and psychophysiological reactivity to a multitude of environmental stimuli [5,6] derived from physical, internal and external, social, or sensory environments (e.g., social experiences, crowds, thoughts, feelings, vitality, intake, bodily pain, etc.) [7] (Homberg et al., 2016). This trait presumably has a strong genetic component, although it may require the presence of particular environmental influences for its expression [5].

Most studies on SPS refer to the description of the characteristics present in people with high sensitivity considered as a single category. However, some authors [8,9,10] categorize SPS into low, medium, and high levels, thus favoring a better identification of the different ways of processing and retrieving information in the brain since variations in emotional response may occur depending on the level of sensitivity. In this regard, there are a greater number of studies focusing on high and low sensitivity [11]. They show that at a cognitive level, people with high sensitivity, compared to those with low sensitivity, use deeper information processing strategies, analyzing in detail before acting. In addition, they have a greater awareness of all the subtleties of the environment, quickly activating the autonomic arousal response.

In explaining behavioral differences in people with SPS, some studies based on Reinforcement Sensitivity Theory [12] report that people with high sensitivity show BIS (behavioral inhibition system) functioning [13,14] and adopt a pause-to-check survival strategy, i.e., they observe by taking longer to make decisions and to process before initiating an action [2]. In contrast, other studies highlight that both the BIS, which responds to novel punishing stimuli or negative events, and the BAS (behavioral activation system), which responds to novel rewarding and non-punishing stimuli, may function together in people with SPS [9,10,15], although there is some consensus that people with high SPS use the strategy of avoidance or withdrawal from potentially threatening situations in an attempt to protect mental health.

This tendency to avoid or withdraw from situations corresponds to the risk or harm avoidance dimension of Cloninger’s (1987) [16] psychobiological model of personality. The tendency to inhibit behaviors that could lead to punishment, in the form of concern about emotional reactivity when processing positive and negative experiences more deeply, would explain the maintenance of emotional distress, from anxiety and sadness to psychological disorders such as dysthymia [17].

Previous studies report the relationship between SPS and the occurrence of psychological [18,19,20,21] and affective-emotional disorders [22], highlighting the relationship with the conscientiousness personality trait, as well as perceived stress [23] and post-traumatic stress [24]. In general, SPS is related to factors associated with inadequate stress management, including difficulty in managing emotions and coping in different contexts, such as at work [23,25], as well as burnout and compassion fatigue [26].

Therefore, it is important for people with SPS to be able to identify their trait, recognize it, and develop appropriate stress-coping strategies in order to achieve self-care and health [27]. Furthermore, the tendency to behave using different coping strategies in the face of stress is implicit in personality, which determines how an individual will adapt to various environments. However, despite the importance given to internal and external stimuli, as well as the difficulty in regulating emotions [28], there are not many previous studies that determine the influence of adaptive and maladaptive coping strategies on quality of life, through indicators of mental (anxiety and depression) and physical (vitality) health and functioning in their lives in different contexts (emotional role functioning). In this sense, contexts that favor the use of successful stress-coping strategies are related to the presence of positive consequences for mental health [24].

In view of the above, this study has three objectives:

(1) To determine gender differences in SPS and its dimensions (sensitivity to overstimulation, aesthetic sensitivity, low sensory threshold, fine psychophysiological discrimination, and harm avoidance), personality traits (neuroticism, extraversion, and conscientiousness), coping strategies, and quality of life through health indicators (mental health, vitality, and emotional role functioning). Similarly, whether there are differences depending on the levels of SPS (low, medium, and high sensitivity) in the variables under study;
(2) To find out the relationship between SPS, personality traits, and coping strategies with quality of life;
(3) To determine the predictive value of personality traits and coping strategies on indicators of health-related quality of life in people with high sensitivity.

## 2. Materials and Methods

### 2.1. Participants

The sample consisted of 10,525 adults, 1741 men and 8784 women, with a mean age of 33.61 (SD = 11.40) years (range 18–80 years). The sample was collected in Spain and was recruited in a community context. Sampling was conducted by convenience and by accessibility.

Participants fulfilled the following inclusion criteria: (a) minimum age of 18 years, (b) filled in all data or the test battery appropriately, and (c) expressly accepted the informed consent. Sample characteristics are presented in Table 1.

### 2.2. Procedure

This was a prospective cross-sectional survey study. The research team was made up of university professors with extensive research careers and professional experts in high-sensitivity persons. The procedure included the following steps: (a) The participants were contacted through different associations of Professionals of High Sensitivity and different Universities with which the research team collaborates. These professionals, in turn, disseminated the research through their social networks and by e-mail, providing the online link for access to participate in the research, and (b) the anonymous online tests were taken, which took 15 to 20 min, through an online application. After reading a brief introduction with the study objectives, main characteristics, and purpose, participants agreed to participate subject to the conditions of the research. Then, they proceeded to the tests, which were always presented in the same order.

Participation was voluntary and anonymous, and no compensation of any kind was received for taking part. All participants signed their informed consent. The participants could drop out of this study at any time. Appropriate measures were taken to safeguard the information in compliance with Spanish Organic Law 3/2018 on data protection and guarantee of digital rights. In addition, this study was performed following the code of ethics of the World Medical Association [29], and it had the institutional approval of the University where this study was developed.

### 2.3. Data Analysis

The assumption of normality was tested using Kolmogorov–Smirnov, and the assumption of homoscedasticity was tested using the Levene Statistic. Sensitivity of sensory processing (SPS) was not adjusted to normal; therefore, non-parametric tests were used.

Descriptive statistics were calculated for all variables (i.e., means and standard deviations for continuous variables and percentages for categorical variables). Chi-square was calculated to assess the significance of the associations among the demographic variables.

According to the above, the Kruskal–Wallis (H) and Mann–Whitney (U) were used to test for the existence of significant differences in the scores of HSPS-S (SPS total and its dimensions), NEO-FFI (neuroticism, extraversion, and conscientiousness), CSI (problem solving, cognitive restructuring, problem avoidance, wishful thinking, emotional expression, social support, social withdrawal, and self-criticism) and SF-36 (mental health, vitality, and emotional role functioning) among women and men. Similarly, analysis of variance (ANOVA) was applied to examine the differences in function of the levels of SPS (low, medium, and high).

Trends and associations among variables were evaluated using Pearson’s correlation analyses. Cohen’s d was calculated using the Lipsey and Wilson method (2001) [30]. Effect size values were <0.30, 0.30–0.50, and >0.50 as small, medium, and large sizes, respectively. To determine the percentage of participants at each sensitivity level (SPS), a new category variable was created based on the criteria: LL (low level): Percentile < 34 (Total High sensitivity person Scale-HSPS-S: Men = 140, Women = 151); ML (medium level): Percentile 34 a 66 (Total HSPS-S: Men = 141–159, Women = 152–167); HL (high level): Percentile > 66) (Total HSPS-S: Men ≥ 160, Women ≥ 168). Linear regressions were performed to determine the risk and protective factors.

Cronbach’s alpha coefficients were used to estimate reliability. Acceptable internal consistency was estimated using those values > 0.80. A *p*-value of less than 0.05 was considered to indicate statistical significance. All statistical analyses were performed using the Statistical Package for Social Sciences (Version 26.0 for Windows).

### 2.4. Measures

#### 2.4.1. High-Sensitivity Person Scale (HSPS)

The HSPS [13], Spanish adaptation [17], is a self-report to identify the highly sensitive person. It consists of 27 direct items with 7 response options on a Likert-type scale (1 = strongly disagree/7 = strongly agree) (range: 27–189). Higher scores indicate a higher degree of sensory sensitivity. In their original research, Aron and Aron (1997) [13] found internal consistency scores of α = 0.87 and α = 0.85. In the Spanish version [17], Cronbach’s α for the subscales, respectively, were as follows: sensitivity overstimulation (SOS) (feeling overwhelmed by both external and internal demands) (α = 0.87), aesthetic sensitivity (AES) (awareness of the aesthetics of the environment) (α = 0.79), low sensory threshold (LST) (sensory discomfort from overstimulation) (α = 0.82), fine psychophysiological discrimination (FPD) (discrimination against subtleties or physical/physiological sensitivity in reaction to internal stimuli) (α = 0.57), and hard avoidance (HA) (controlled avoidance of harm) (α = 0.68). For the total HSPS-S, α = 0.92.

#### 2.4.2. Personality Inventory (NEO-FFI)

The NEO-FFI (NEO Five Factor Inventory [31], Spanish adaptation [32]) assesses normal personality according to the Big Five model. It consists of 60 items distributed in 5 factors or basic dimensions, with 12 items each: neuroticism, extraversion, openness, agreeableness, and conscientiousness. It has 5 response options on a Likert-type scale (0 = strongly disagree/4 = strongly agree) (range: 0–48), with each factor being assessed independently. It has good psychometric measures, and Cronbach’s alpha coefficients range from 0.82 to 0.90 in the original version [31]. In this current study, they range between 0.73 and 0.85. The factors used in this study are neuroticism (N), extraversion (E), and conscientiousness (C) (α = 0.79; α = 0.84; α = 0.83; respectively).

#### 2.4.3. Coping Strategies Inventory (CSI)

The CSI (Coping Strategies Inventory [33], Spanish adaptation [34]) assesses coping strategies along two axes: engagement-focused strategies and disengagement strategies. Additionally, the two objective categories of coping, problem-focused and emotion-focused, are positioned on a second axis. From this combination, the eight strategies are categorized into the following: problem-focused engagement includes problem solving (PS) and cognitive restructuring (CR), problem-focused disengagement encompasses problem avoidance (PA) and wishful thinking (WT) strategies, emotion-focused engagement consists of emotional expression (EE) and social support (SS) strategies, and emotion-focused disengagement comprises social withdrawal (SW) and self-criticism (SC). It consists of 40 direct items, 5 for each strategy, with 5 response options on a Likert-type scale (1 = never/5 = almost always) (range: 5–40). Internal consistency coefficients were between 0.63 and 0.89 in the Spanish adaptation [34]. In this current study, they range from 0.70 (problem avoidance) to 0.88 (self-criticism). Eight coping strategies were used in this study.

#### 2.4.4. Short Form Health Survey (SF-36)

The SF-36 (Short Form Health Survey [35], Spanish adaptation [36]) assesses health-related quality of life in the general population and specific populations (general population and patients with other health conditions). It consists of 36 items, distributed in 8 scales: general health, mental health, physical role, emotional role, physical function, social function, and bodily pain. It presents several response options on a Likert-type scale, with each subscale rated independently (range: 0–100). Scores close to 0 indicate poor quality of life, and close to 100 indicate excellent. Psychometric measures in previous studies are good [36]. In this current study, three scales have been used: domains of mental health (MH: feelings of happiness, calmness, and tranquility vs. feelings of anxiety and depression) (α = 0.85), vitality (V: feeling dynamic and energetic vs. tired and/or exhausted) (α = 0.84), and emotional role functioning (ERF: functioning in different domains of daily life due to emotional problems) (α = 0.92).

## 3. Results

### 3.1. Results of the Comparison between Men and Women

For Objective 1, the sample was divided according to gender: 1741 men (16.5%) and 8784 women (83.5%). In demographic characteristics, differences (*p* = 0.000) were observed in age (t10522 = −4.031), age range (χ^2^ (4) = 41.540), marital status (χ^2^ (5) = 51.954), and educational level (χ^2^ (4) = 84.974) (Table 1).

#### 3.1.1. Women Score Higher on SPS

In psychological and health-related quality of life characteristics (Table 2), women had higher scores on total sensory processing sensitivity. In all five dimensions, personality traits, higher tendencies to use problem-focused adaptive coping strategies (problem solving and cognitive restructuring) on emotion, emotional expression, and social support, problem-focused maladaptive strategies (wishful thinking) on emotion, and emotion-focused maladaptive strategies (self-criticism), as well as lower scores on health. Men had higher scores on the maladaptive coping strategies problem avoidance: problem avoidance as a problem-focused maladaptive coping strategy and social withdrawal as an emotion-focused disengagement.

#### 3.1.2. Differences between Men and Women in SPS

Differences between men and women were observed in sensory-processing sensitivity regarding total and its dimensions (*p* = 0.000), in the personality traits of neuroticism, including conscientiousness (*p* = 0.000) and extraversion (*p* = 0.045), in emotion-focused disengagement coping strategy of social withdrawal (*p* = 0.000), in problem-focused disengagement strategies, including problem avoidance and wishful thinking (*p* = 0.000), and in emotion-focused engagement strategies of emotional expression and social support (*p* = 0.000). In health-related quality of life, there were differences in all three indicators studied (mental health, vitality, and emotional role functioning) (*p* = 0.000). All effect sizes were small.

There were no differences in the emotion-focused disengagement strategy of self-criticism (*p* = 0.212) and in the problem-focused engagement strategies of problem solving (*p* = 0.337) and cognitive restructuring (*p* = 0.797).

**Figure 1:**
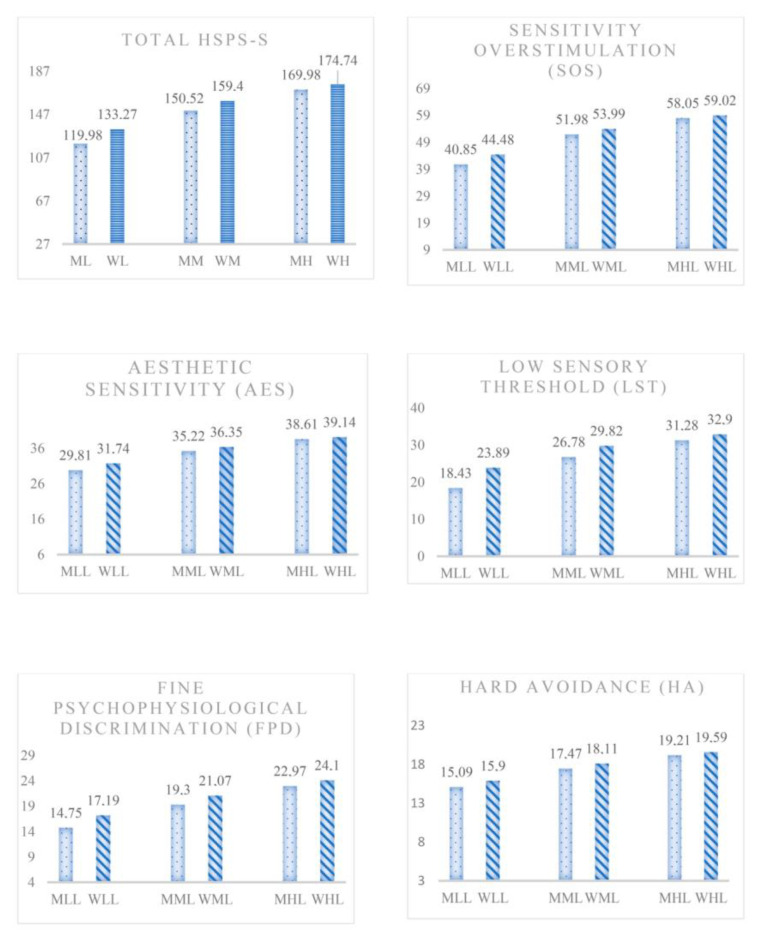
Mean scores by gender and by SPS levels (low, medium, and high) on the HSPS-S.

The sample was then categorized according to the level of SPS (low, medium, and high) (Figure 1 and Table 3). In personality traits, differences between levels were observed in the variables studied (*p* = 0.000). In pairwise comparisons, significant differences were obtained in neuroticism, extraversion, and conscientiousness, between low and medium (*p* = 0.000), low and high (*p* = 0.000), and medium and high sensitivity (*p* = 0.000), in both males and females. In the pairwise comparisons between low and medium sensitivity, medium and high sensitivity, and low and high sensitivity, in both sexes, the effect sizes were small. In contrast, in neuroticism in the low–high sensitivity comparison, effect sizes were medium, in both males and females. It was observed that mean scores were higher on personality traits at the high level of sensitivity in both sexes, and in women, they were higher than in men. In other words, in general, high scores in sensory processing sensitivity are accompanied by high scores in the personality traits studied.

In coping strategies, differences were observed between levels (*p* = 0.000), except for social support in men (*p* = 0.098). In pairwise comparisons, in problem-focused engagement strategies, there were significant differences in problem solving and cognitive restructuring between low–medium (*p* = 0.005; *p* = 0.004), low–high, and medium–high (*p* = 0.000; *p* = 0.000), in women and in men between low–high (*p* = 0.002; *p* = 0.047), low–medium (*p* = 0.001) in cognitive restructuring, and medium–high (*p* = 0.000) in problem solving. Effect sizes were small. There were no differences in men between low–medium in both strategies (*p* = 0.744; *p* = 0.678).

In emotion-focused engagement strategies, emotional expression and peer comparisons were significant (*p* = 0.000) in both sexes; in men between low–medium, it was *p* = 0.002. In social support, in women, there was between low–medium (*p* = 0.020) and low–high sensitivity (*p* = 0.005). Effect sizes were small. There were no differences in social support in men between low–medium (*p* = 0.653), low–high sensitivity (*p* = 0.341), and medium–high sensitivity (*p* = 0.065), nor in women between medium–high sensitivity (*p* = 0.632). It was observed that mean scores were generally higher at the high sensitivity level in both sexes, and in women, they were higher than in men. In cognitive restructuring, they were similar in both sexes.

Regarding problem-focused disengagement strategies, in wishful thinking, pairwise comparisons were significant (*p* = 0.000) in both sexes. In problem avoidance, there were differences in men between low–medium (*p* = 0.001) and low–high sensitivity (*p* = 0.000) and in women between low–medium (*p* = 0.004), low–high (*p* = 0.000), and medium–high (*p* = 0.034). There were no differences in men between medium and high sensitivity (*p* = 0.180). Effect sizes were small, except in wishful thinking, between low and high sensitivity in men, with medium effect sizes.

In emotion-focused disengagement strategies, self-criticism, social withdrawal, and peer comparisons were significant (*p* = 0.000) in both sexes. In self-criticism, between medium and high sensitivity, in men, it was *p* = 0.008. Effect sizes were small. It was observed that mean scores were higher at the high level of sensitivity in both sexes: in women, scores were higher in self-criticism and wishful thinking; in men, scores were higher in problem avoidance and social withdrawal, indicating a greater tendency of men towards avoidance strategies.

In pairwise comparisons on health indicators, pairwise comparisons were significant in both women and men (*p* = 0.000), except in men between medium and high sensitivity in mental health (*p* = 0.002), vitality (*p* = 0.013), and emotional role functioning (*p* = 0.001). Effect sizes were small, except for mental health in men, between low and high sensitivity, with medium effect sizes.

It is noteworthy that in the SF-36 general health assessment, 13.7% (M = 1.8% vs. W = 11.9%) of participants in the low SPS level, 19.2% (M = 2.7% vs. W = 16.5%) in the medium level, and 26.6% (M = 3.2% vs. W = 23.6%) of participants in the high level perceived their general health as fair/bad or poor.

### 3.2. Relationship between SPS, Selected Psychological Variables, Namely Personality Traits and Coping Strategies, with Health Indicators

Regarding Objective 2, in the relationship between SPS, certain psychological variables, namely personality traits (neuroticism, extraversion, and conscientiousness) and coping strategies, with health indicators (mental health, vitality, and emotional role functioning), the results showed significant relationships in all three indicators (*p* = 0.000): in emotional role functioning with social support, it was *p* = 0.001; with problem avoidance, it was *p* = 0.004 in vitality with all variables as well as mental health (*p* = 0.000), except for emotional expression (*p* = 0.573). Effect sizes were small, except for self-criticism and wishful thinking, which were medium in all three health indicators, for extraversion in mental health and vitality, and for cognitive restructuring and social withdrawal in mental health. In neuroticism, effect sizes were high in mental health, vitality, and emotional role functioning (Table 4).

### 3.3. Predictive Value of Personality Traits and Coping Strategies on Health Indicators in Highly Sensitive Persons

For Objective 3, to determine the predictive value of personality traits and coping strategies on health indicators in highly sensitive persons, linear regression analyses were used using the stepwise method. For a comprehensive analysis of high sensitivity, participants categorized in the medium and high levels of the HSPS-S (n = 7029) were selected.

From the proposed models, the percentages of variance explained in the health indicators were 47.7% in mental health (F = 915.377, *p* = 0.000), 32.7% in vitality (F = 567.651, *p* = 0.000), and 25.1% in emotional role functioning (F = 392.360, *p* = 0.000) (Table 5 part a).

In the model for mental health (β = 77.266; t = 52.930; *p* = 0.000) (Table 5 part b), the results obtained indicated that personality traits would explain 43.8% (specifically, 41.1% neuroticism, 2.6% extraversion, and 0.1% conscientiousness) and coping strategies would explain 2.9% (specifically, 1.7% maladaptive coping strategies). In this proposed model, in people with medium–high sensory sensitivity, neuroticism (*p* = 0.000) and using maladaptive coping strategies, such as self-criticism, wishful thinking, and social withdrawal (*p* = 0.000), would act as risk factors for mental health, which explains 43.5% of the total weighted variance. On the other hand, using problem-focused adaptive coping strategies, cognitive restructuring (*p* = 0.000), as well as having extraversion (*p* = 0.000), and conscientiousness (*p* = 0.007) personality traits would act as a protective factor for mental health, explaining 3.9% of the total variance. 

Regarding the health indicator of vitality, in the model (β = 45.000; t = 25.498; *p* = 0.000), the results indicated that personality traits would explain 31.2% (specifically, 22% neuroticism, 8.8% extraversion, and 0.4% conscientiousness) and coping strategies 1.5%. In this model, neuroticism (*p* = 0.000), and maladaptive coping strategies, such as self-criticism (*p* = 0.004) and wishful thinking (*p* = 0.000), would act as risk factors for vitality, together explaining 23.4% of the total weighted variance. On the other hand, using adaptive coping strategies focused both on the problem, such as cognitive restructuring (*p* = 0.000), and on the emotion, such as emotional expression (*p* = 0.000), problem-focused maladaptive coping strategies, such as low tendency avoidance problems (*p* = 0.024), as well as presenting extraversion and conscientiousness personality traits (*p* = 0.000), would act as a protective factor in vitality, explaining 12.8% of the total variance. 

Finally, in the model proposed to determine the weight of the predictor variables studied on the emotional role functioning criterion variable (β = 102.654; t = 36.484; *p* = 0.000), the obtained results indicated that personality traits would explain 22.6% of the total variance and coping strategies 2.6%. In this model, neuroticism (*p* = 0.000) and maladaptive coping strategies, such as wishful thinking and social withdrawal (*p* = 0.000), would act as risk factors on emotional role functioning, together explaining 23.1% of the total weighted variance. On the other hand, having personality traits of extraversion and conscientiousness (*p* = 0.000) would act as a protective factor on emotional role functioning, explaining 1.31% of the total variance. 

## 4. Discussion

The general objective of this study conducted with a representative population of people with sensory processing sensitivity is to determine the weight of personality traits and coping strategies used, on quality of life, by assessing physical and mental health. We aim to determine gender differences in SPS and its dimensions, personality traits, coping strategies, and quality of life through health indicators to find out if there are differences depending on the levels of SPS in the variables under study. We also aim to find out the relationship between SPS, personality traits, and coping strategies with quality of life and to determine the predictive value of personality traits and coping strategies on indicators of health-related quality of life in people with high sensitivity. Previous studies conducted with highly sensitive people have shown the relationship between this personality trait and others, such as neuroticism and extraversion [1,14,37,38,39]. In contrast, few previous studies have been conducted either with coping strategies in this population despite their importance having been reported [40] or with the personality trait of conscientiousness, hence the special interest of the authors of this paper.

First of all, an analysis was performed as to whether men and women showed differences in the variables studied. The findings obtained reported, on the one hand, a greater SPS overall, and in the different dimensions, they reported a greater presence of the three personality traits studied, a more frequent tendency to use maladaptive and adaptive coping strategies focused on the problem and a poorer quality of life, based on health indicators. Men, on the other hand, tended to use emotion-oriented avoidant coping strategies, such as problem avoidance and social withdrawal, more frequently.

In this respect, the findings are in line with those of Hajek et al. (2020) [41]. These authors report, from their systematic review, a relationship between high extraversion and high neuroticism with higher use of health devices, with neuroticism being the trait that explains a lower use of health behaviors and poorer coping with stress. As for trait conscientiousness, on the other hand, some studies relate this trait to health-promoting behaviors [42]. In relation to gender, previous studies report higher scores among women in SPS [43] and neuroticism [44], especially anxiety [31].

On the other hand, it was observed that high levels of sensory processing sensitivity were also accompanied by higher scores in the personality traits of neuroticism and conscientiousness, except extraversion, in all coping strategies, with the exception of problem avoidance and social withdrawal and by worse mental health, emotional role functioning, and vitality, in both women and men. Therefore, the presence of high sensitivity is manifested by a higher presence of the personality traits of neuroticism and conscientiousness, lower extraversion, higher tendency to use maladaptive coping strategies, and higher impairment in health-related quality of life. These findings, in line with those of Lionetti et al. (2018) [9], confirm the need to categorize people with SPS into levels, for a better understanding of how they process and retrieve information in the brain. On the other hand, contexts that favor the use of successful stress-coping strategies are related to the presence of positive mental health outcomes [24].

The second objective was to determine the relationship between the different variables with indicators of health-related quality of life. The findings revealed significant relationships in mental health, vitality, and emotional role functioning, with certain psychological variables, specifically coping strategies and personality traits. Notably, the use of maladaptive coping strategies (wishful thinking, self-criticism, and social withdrawal) was related to poorer quality of life, i.e., poorer mental health and emotional role, and lower vitality, i.e., physical health. In addition, higher levels of sensory-processing sensitivity and neuroticism and lower levels of extraversion and conscientiousness are related to poorer health-related quality of life. This leads us to affirm that an association exists between stimuli from the internal or external environment of the person with SPS, be they physical, social, or sensory stimuli [2], with the way of coping with work stress and the wellbeing of the person with SPS. In this sense, the relationship between SPS and personal wellbeing [45] is evident in behaviors that support adaptation to the environment. In particular, deep information processing in people with SPS favors their adaptation to possible threats from the environment [5,46].

The third objective was to determine the predictive value of personality traits and coping strategies in health indicators (mental health and vitality) in people with medium–high sensitivity and their impact on personal and occupational functioning (emotional role). It is noteworthy that with respect to the three health-related study variables, neuroticism is the trait with the highest predictive power, acting as a risk factor. These findings are congruent with previous research in which SPS is related to temperament and personality traits, such as neuroticism, associated with greater reactivity to environmental influences [3,39]. In terms of coping strategies, social withdrawal (in the case of mental health and emotional role functioning) and self-criticism (in the case of mental health and vitality) act as risk factors, which leads us to consider the importance of achieving mental health or maintaining it, and vitality and the emotional role act as factors associated with SPS. Therefore, interaction with negative environments may increase the risk of poor adaptation, with negative outcomes for general health and mental [28,47] and physical health [28,48], as in our study, where 25% (45% if considering medium–high sensitivity) approximately perceive their general health as poor/regular or bad.

In contrast, extraversion and conscientiousness personality traits, as well as the use of adaptive coping strategies, such as cognitive restructuring, act as a protective factor in mental health, vitality, and emotional role functioning. Furthermore, problem avoidance and emotional expression exert a protective role in vitality and emotional role functioning, respectively. In this sense, previous studies indicate the relationship between the way of managing desirable life experiences and subjective wellbeing [1]. This leads us to think of SPS as central to certain personality variables [49], such as conscientiousness, as well as the development of problem-solving and cognitive-restructuring skills [4], with the aim of promoting positive environments for the optimal development of people with SPS [40,50,51]. Therefore, incorporating strategies for the appropriate management of sensitivity and emotional reactivity into quality-of-life prevention programs would have positive consequences for mental health [24] by favoring a reduction in the presence of psychological disorders, including dysthymic disorders [17], anxiety, and depression [25,37,52].

In summary, it has been found that personality traits and coping strategies through health indicators (mental, vitality, and emotional role functioning) exert an influence on the quality of life in people with sensory processing sensitivity.

## 5. Conclusions

Based on the study objectives, the findings show that women show greater sensory-processing sensitivity (with an average of 8.92 points higher than men), a greater presence of the personality traits studied, a more frequent tendency to use maladaptive and adaptive coping strategies focused on the problem, as well as a worse quality of life, based on health indicators. In addition, the presence of high sensitivity is manifested by a greater presence of neuroticism and conscientiousness, lower extraversion, greater tendency to use maladaptive coping strategies, and greater impairment in health-related quality of life. On the other hand, in line with previous studies, neuroticism is the personality trait with the highest predictive power, acting as a risk factor, while extraversion and conscientiousness act as protective factors. In addition, the use of certain maladaptive coping strategies, such as social withdrawal and self-criticism, act as risk factors, whereas the use of adaptive coping strategies, such as cognitive restructuring, problem avoidance, and emotional expression, play a protective role.

These findings highlight the need to develop prevention programs to promote quality-of-life strategies for the appropriate management of sensory processing sensitivity, with special emphasis on mental and physical health (vitality) and emotional role functioning, with the aim of enhancing personal and contextual functioning for people with high sensitivity.

We encourage the international research community that, in future studies, future research should continue to examine the associations of SPS with quality of life, as this may help the understanding of SPS in relation to health improvement.

With regard to possible limitations of this study, those specific to the online evaluation should be mentioned, among which is the impossibility of controlling certain strange variables that could have interfered with completing the questionnaires. Additionally, the absence of previous research, as the SPS is still in its early stages [5]. Similarly, although the sample was very large and representative, people without internet access were not evaluated. For future work, it would be interesting to extend the evaluation to include the face-to-face modality, especially in those studies that test the possible prevention programs derived from this work, with a view to guaranteeing equal opportunities in the population under study.

## Figures and Tables

**Table 1:** Participant’s characteristics (n = 10,525).

		Men (n = 1741)		Women (n = 8784)	
		n	%	n	%
Age					
	Mean (Range: 18–80)	34.63	16.5	33.42	83.5
	SD	12.41		11.20	
Age group					
	≤30	786	7.5	4030	38.3
	31–40	438	4.2	2393	22.7
	41–50	303	2.9	1650	15.7
	51–60	160	1.5	587	5.6
	≥61	54	0.5	124	1.2
Marital status					
	Single	997	9.5	4263	40.5
	With partner	245	2.3	1676	15.9
	Married	303	2.9	1826	17.3
	Divorced	141	1.3	712	6.8
	Widowed	4	0.0	38	0.4
	Not specified	51	0.5	269	2.6
Educational level					
	College	895	8.5	5316	50.4
	High school	640	6.1	2827	26.9
	Secondary	150	1.4	530	5.0
	Primary	50	0.5	101	1.0
	Without studies	6	0.1	10	0.1

**Table 2:** Mean gender differences.

		Total (N = 10,525)	Men (n = 1741)	Women (n = 8784)						
		M (SD)	M (SD)	M (SD)	U Mann–Whitney Comparison of Mean		*p*-Value	df	Cohen d	95% CI
Sensory-processing sensitivity (HSPS-S)										
	Total SPS	154.35 (22.56)	146.90 (24.16)	155.82 (21.93)	5,767,688		0.000 ***	8.917	−0.39 s	153.91 to 154.78
	SOS	52.16 (8.91)	50.29 (9.60)	52.53 (8.72)	6,522,625		0.000 ***	2.239	−0.24 s	51.99 to 52.33
	AES	35.55 (5.39)	34.55 (5.75)	35.75 (5.29)	6,628,438		0.000 ***	1.201	−0.22 s	35.45 to 35.66
	LST	28.38 (5.85)	25.84 (6.49)	28.89 (5.57)	5,339,923		0.000 ***	3.042	−0.50 s	28.27 to 28.49
	FPD	20.49 (4.56)	18.98 (4.94)	20.79 (4.42)	6,007,838.50		0.000 ***	1.810	−0.38 s	20.40 to 20.58
	HA	17.76 (2.97)	17.24 (3.15)	17.86 (2.92)	6,678,179.50		0.000 ***	0.625	−0.20 s	17.70 to 17.82
Personality traits (NEO-FFI)										
	N	26.19 (5.60)	25.18 (6.33)	26.38 (5.91)	6,790,741		0.000 ***	1.200	−0.20 s	26.09 to 26.30
	E	23.58 (6.68)	23.27 (7.02)	23.65 (6.61)	7,414,177		0.016 *	0.380	−0.06 s	23.46 to 23.71
	C	30.20 (6.63)	29.01 (6.88)	30.44 (6.55)	6,747,961.50		0.000 ***	1.422	−0.21 s	30.07 to 30.33
Coping strategies (CSI)										
	PS	12.72 (4.12)	12.58 (4.36)	12.75 (4.07)	7,535,438		0.337	0.171	−0.04 s	12.64 to 12.80
	SC	12.03 (5.01)	11.91 (4.89)	12.06 (5.03)	7,502,218.50		0.212	0.153	−0.03 s	11.94 to 12.13
	EE	10.64 (4.75)	8.86 (4.60)	11.00 (4.70)	5,638,580.50		0.000 ***	2.139	−0.46 s	10.55 to 10.73
	WT	13.40 (4.91)	12.54 (4.98)	13.57 (4.88)	6,723,761.50		0.000 ***	1.029	−0.21 s	13.30 to 1.302
	SS	9.69 (3.89)	8.85 (4.67)	9.86 (4.91)	6,770,217		0.000 ***	1.003	−0.21 s	9.60 to 9.78
	CR	11.12 (3.90)	11.08 (3.88)	11.13 (3.90)	7,616,698.50		0.797	0.053	−0.01 s	11.05 to 11.20
	PA	5.30 (3.58)	5.84 (3.68)	5.20 (3.55)	84,447,475		0.000 ***	−0.643	0.18 s	5.23 to 5.37
	SW	9.04 (4.38)	9.98 (4.51)	8.87 (4.33)	8,750,097		0.000 ***	−1.114	0.25 s	8.97 to 9.14
Short Form Health Survey (SF36)										
	MH	49.98 (16.72)	51.63 (17.94)	49.65 (16.45)	8,092,909.50		0.000 ***	−1.978	0.11 s	49.66 to 50.30
	V	47.34 (17.91)	50.99 (18.83)	46.61 (17.64)	8,658,266		0.000 ***	−4.377	0.24 s	46.99 to 47.68
	ERF	61.47 (21.22)	64.01 (21.20)	60.96 (21.20)	8,096,128.50		0.000 ***	−3.052	0.14 s	61.06 to 61.87

*** *p* ≤ 0.001, * *p* ≤ 0.05. Cohen d effect size: s = small magnitude ratio: <0.30. Total SPS = Total HSPS-S; SOS = Sensitivity to overstimulation; AES = Aesthetic sensitivity; LST = Low sensory threshold; FPD = Fine psychophysiological discrimination; HA = Harm avoidance; N = Neuroticism; E = Extraversion; C = Conscientiousness; PS = Problem solving; SC = Self-criticism; EE = Emotional expression; WT = Wishful thinking; SS = Social support; CR = Cognitive restructuring; PA = Problem avoidance; SW = Social withdrawal; MH = Mental health; V = Vitality; ERF = Emotional role functioning.

**Table 3:** Mean gender difference according to sensorial processing sensitivity level (N = 10,525).

		HSPS-S Men ^a^ (n = 1741)			HSPS-S Women ^b^ (n = 8784)			Comparison of Mean		Paired Comparison of Mean (U) ^h^								
		LL ^c^	ML ^d^	HL ^e^	LL	ML	HL			LL ^c^–ML ^d^			LL ^c^–HL ^e^			ML ^d^–HL ^e^		
		M (SD)	M (SD)	M (SD)	M (SD)	M (SD)	M (SD)	H ^g^	*p*-Value		*p*	Cohen d^f^		*p*-Value	Cohen d		*p*-Value	Cohen d
Personality traits (NEO-FFI)																		
	N	22.32	25.83	27.38	24.57	26.47	28.11	207.97	0.000 ***	^a^	0.000 ***	−0.58 s	^a^	0.000 ***	−0.84 m	^a^	0.000 ***	−0.27 s
		(6.35)	(5.73)	(5.74)	(5.74)	(5.59)	(5.87)	523.31	0.000 ***	^b^	0.000 ***	−0.33 s	^b^	0.000 ***	−0.61 m	^b^	0.000 ***	−0.29 s
	E	25.56	22.74	21.51	25.58	23.46	21.90	102.88	0.000 ***	^a^	0.000 ***	0.41 s	^a^	0.000 ***	0.58 s	^a^	0.000 ***	0.18 s
		(7.19)	(6.47)	(6.77)	(6.73)	(6.30)	(6.27)	450.36	0.000 ***	^b^	0.000 ***	0.32 s	^b^	0.000 ***	0.56 s	^b^	0.000 ***	0.25 s
	C	28.22	28.29	30.51	29.41	30.17	31.74	42.37	0.000 ***	^a^	0.000 ***	−0.27 s	^a^	0.000 ***	−0.34 m	^a^	0.000 ***	−0.34 s
		(7.33)	(6.94)	(6.08)	(6.59)	(6.44)	(6.41)	195.64	0.000 ***	^b^	0.000 ***	−0.12 s	^b^	0.000 ***	−0.36 s	^b^	0.000 ***	−0.24 s
Coping strategies (CSI)																		
	PS	12.39	12.20	13.14	12.23	12.53	13.49	16.72	0.001 **	^a^	0.850	−0.04 s	^a^	0.002 **	−0.17 s	^a^	0.000 ***	−0.22 s
		(4.43)	(4.22)	(4.39)	(4.04)	(3.93)	(4.12)	156.79	0.000 ***	^b^	0.005 **	−0.07 s	^b^	0.000 ***	−0.31 s	^b^	0.000 ***	−0.24 s
	SC	10.24	12.38	13.09	10.72	12.01	13.44	104.91	0.000 ***	^a^	0.000 ***	−0.44 s	^a^	0.000 ***	−0.59 s	^a^	0.008 **	−0.15 s
		(4.77)	(4.56)	(4.87)	(4.90)	(4.86)	(4.95)	436.03	0.000 ***	^b^	0.000 ***	−0.26 s	^b^	0.000 ***	−0.55 s	^b^	0.027 *	−0.29 s
	EE	7.79	8.63	10.14	10.32	10.93	11.74	80.81	0.000 ***	^a^	0.002 **	−0.19 s	^a^	0.000 ***	−0.51 s	^a^	0.000 ***	−0.33 s
		(4.42)	(4.39)	(4.68)	(4.59)	(4.57)	(4.84)	125.82	0.000 ***	^b^	0.000 ***	−0.13 s	^b^	0.000 ***	−0.30 s	^b^	0.000 ***	−0.17 s
	WT	10.71	12.58	14.30	12.22	13.53	14.95	152.81	0.000 ***	^a^	0.000 ***	−0.38 s	^a^	0.000 ***	−0.74 m	^a^	0.000 ***	−0.37 s
		(5.02)	(4.60)	(4.66)	(4.91)	(4.75)	(4.58)	476.35	0.000 ***	^b^	0.000 ***	−0.27 s	^b^	0.000 ***	−0.57 s	^b^	0.000 ***	−0.30 s
	SS	8.80	8.56	9.19	10.09	9.77	9.71	4.65	0.098	^a^	0.653	0.05 s	^a^	0.341	−0.08 s	^a^	0.065	−0.13 s
		(4.47)	(4.53)	(4.98)	(4.94)	(4.73)	(5.04)	8.97	0.011 *	^b^	0.020 *	0.07 s	^b^	0.005 **	0.08 s	^b^	0.632	0.01 s
	CR	10.96	10.77	11.50	10.81	11.08	11.50	9.35	0.009 **	^a^	0.314	0.05 s	^a^	0.046 *	0.14 s	^a^	0.003 **	−0.18 s
		(3.72)	(3.77)	(4.08)	(3.82)	(3.81)	(4.02)	41.99	0.000 ***	^b^	0.004 **	−0.07 s	^b^	0.000 ***	−0.17 s	^b^	0.000 ***	−0.11 s
	PA	6.36	5.70	5.46	5.47	5.17	4.94	22.06	0.000 ***	^a^	0.001 **	0.18 s	^a^	0.000 ***	0.24 s	^a^	0.180	0.06 s
		(3.63)	(3.65)	(3.72)	(3.44)	(3.46)	(3.73)	49.94	0.000 ***	^b^	0.004 **	0.09 s	^b^	0.000 ***	0.15 s	^b^	0.034 *	0.06 s
	SW	8.88	10.18	10.88	7.83	8.96	9.82	58.78	0.000 ***	^a^	0.001 **	−0.29 s	^a^	0.000 ***	−0.44 s	^a^	0.017 *	−0.16 s
		(4.56)	(4.30)	(4.44)	(4.24)	(4.16)	(4.37)	316.09	0.000 ***	^b^	0.001 **	−0.27 s	^b^	0.000 ***	−0.46 s	^b^	0.000 ***	−0.20 s
Short Form Health Survey (SF36)																		
	MH	58.69	49.80	46.43	54.21	49.47	45.28	142.32	0.000 ***	^a^	0.000 ***	0.48 s	^a^	0.000 ***	0.70 m	^a^	0.002 **	0.20 s
		(18.05)	(16.91)	(16.58)	(16.53)	(15.70)	(15.89)	416.44	0.000 ***	^b^	0.000 ***	0.17 s	^b^	0.000 ***	0.43 s	^b^	0.000 ***	0.26 s
	V	56.55	49.78	46.66	51.22	46.47	42.15	83.42	0.000 ***	^a^	0.000 ***	0.35 s	^a^	0.000 ***	0.48 s	^a^	0.013 *	0.17 s
		(18.79)	(17.88)	(18.46)	(17.16)	(16.78)	(17.80)	376.02	0.000 ***	^b^	0.000 ***	0.28 s	^b^	0.000 ***	0.52 s	^b^	0.000 ***	0.25 s
	ERF	68.66	63.40	60.01	65.89	61.20	55.79	126.39	0.000 ***	^a^	0.000 ***	0.24 s	^a^	0.000 ***	0.38 s	^a^	0.001 **	0.16 s
		(20.15)	(20.29)	(19.89)	(20.01)	(20.64)	(21.73)	407.15	0.000 ***	^b^	0.000 ***	0.23 s	^b^	0.000 ***	0.48 s	^b^	0.000 ***	0.25 s

*** *p* ≤ 0.001 ** *p* ≤ 0.01 * *p* ≤ 0.05. ^a^ Men; ^b^ Women; ^c^ LL = Low-level SPS; ^d^ ML = Medium-level SPS; ^e^ HL = High-level SPS. Cohen d^f^: effect size: small magnitude ratio: <0.30; mean: between 0.30 and 0.49; high: >0.49. Total SPS = Total HSPS-S: LL (low level): Percentile < 34 [Men = 140 (n = 579), Women = 151 (n = 2917)]; ML (medium level): Percentile 34–66 [(Men = 141–159 (n = 577), Women = 152–167 (n = 2948)]; HL (high level): Percentile > 66 [Men = 160 (n = 585), Women = 168 (n = 2919)]. N = Neuroticism; E = Extraversion; C = Conscientiousness; PS = Problem solving; SC= Self-criticism; EE = Emotional expression; WT = Wishful thinking; SS = Social support; CR = Cognitive restructuring; PA = Problem avoidance; SW = Social withdrawal; MH = Mental health; V = Vitality; ERF = Emotional role functioning. ^g^ Comparison of mean: H = Kruskal–Wallis. ^h^ Paired comparison of mean: U = Mann–Whitney.

**Table 4:** Pearson correlation coefficients of mental health, vitality, and emotional role functioning with other variables (N = 10,525).

		Mental Health	Vitality	Emotional Role Functioning
Sensitivity of sensory processing				
	Total HSPS-S	−0.230 *** (s) 0.000	−0.223 *** (s) 0.000	−0.250 *** (s) 0.000
Personality traits (NEO-FFI)				
	Neuroticism	−0.684 *** (h) 0.000	−0.511 *** (h) 0.000	−0.509 *** (h) 0.000
	Extroversion	0.400 *** (m) 0.000	0.460 *** (m) 0.000	0.220 *** (s) 0.000
	Conscientiousness	0.228 *** (s) 0.000	0.212 *** (s) 0.000	0.248 *** (s) 0.000
Coping strategies (CSI)				
	Problem solving	0.244 *** (s) 0.000	0.241 *** (s) 0.000	0.163 *** (s) 0.000
	Self-criticism	−0.482 ** (m) 0.000	−0.352 *** (m) 0.000	−0.361 *** (m) 0.000
	Emotional expression	−0.005 0.573	0.037 *** (s) 0.000	−0.125 *** (s) 0.000
	Wishful thinking	−0.439 ** (m) 0.000	−0.344 *** (m) 0.000	−0.342 *** (m) 0.000
	Social support	0.159 *** (s) 0.000	0.170 *** (s) 0.000	0.031 ** (s) 0.001
	Cognitive restructuring	0.324 *** (m) 0.000	0.285 *** (s) 0.000	0.133 *** (s) 0.000
	Problem avoidance	0.118 *** (s) 0.000	0.101 *** (s) 0.000	0.028 ** (s) 0.004
	Social withdrawal	−0.332 *** (m) 0.000	−0.281 *** (s) 0.000	−0.250 *** (s) 0.000

** *p* ≤ 0.01, *** *p* ≤ 0.001. Effect size: small magnitude ratio: <0.30; mean: between 0.30 and 0.49; high: >0.49.

**Table 5:** (**a**,**b**) Stepwise multiple linear regression analysis of mental health, vitality, and emotional role functioning (SF-36) (n = 7029).

(**a**)																	
**Mental Health**						**Vitality**						**Emotional Role Functioning**					
**Models**	**R^2^**	**∆R^2^**		**CambR^2^**		**Models**	**R^2^**	**∆R^2^**		**CambR^2^**		**Models**	**R^2^**	**∆R^2^**		**CambR^2^**	
1	0.411	0.411		0.411		1	0.220	0.220		0.220		1	0.213	0.213		0.213	
2	0.447	0.447		0.026		2	0.308	0.308		0.088		2	0.225	0.225		0.013	
3	0.459	0.459		0.012		3	0.315	0.315		0.007		3	0.235	0.234		0.009	
4	0.471	0.471		0.012		4	0.322	0.322		0.007		4	0.239	0.239		0.005	
5	0.476	0.475		0.004		5	0.326	0.326		0.004		5	0.251	0.250		0.011	
6	0.477	0.476		0.001		6	0.327	0.326		0.001		6	0.251	0.250		0.001	
7	0.477	0.477		0.001		7	0.327	0.326		0.000							
R^2^ = 47.7% F = 915.377; *p* = 0.000; df = 7.7021						R^2^ = 32.7% F = 567.651; *p* = 0.000; df = 7.7021						R^2^ = 25.1% F = 392.360; *p* = 0.000; df = 6.7022					
(**b**)																	
	**Mental Health**						**Vitality**						**Emotional Role Functioning**				
	**β**	**Beta**	**t**		***p*-Value**		**β**	**Beta**	**t**		***p*-Value**		**β**	**Beta**	**t**		***p*-Value**
Constant	77.266		52.93		0.000 ***	Constant	45.000		25.50		0.000 ***	Constant	102.654		36.48		0.000 ***
Neuroticism	−1.294	−0.466	−40.05		0.000 ***	Neuroticism	−0.824	−0.271	−20.51		0.000 ***	Neuroticism	−1.821	−0.336	−26.01		0.000 ***
Cognitive restructuring	0.536	0.131	13.73		0.000 ***	Extraversion	0.784	0.284	26.38		0.000 ***	Social withdrawal	−0.879	−0.121	−9.58		0.000 ***
Wishful thinking;	−0.287	−0.084	−7.65		0.000 ***	Cognitive restructuring	0.359	0.080	6.72		0.000 ***	Emotional expression	−0.806	−0.122	−10.46		0.000 ***
Extraversion	0.282	0.112	11.04		0.000 ***	Wishful thinking	−0.332	−0.089	−7.14		0.000 ***	Conscientiousness	0.645	0.134	12.23		0.000 ***
Self-criticism	−0.273	−0.083	−7.07		0.000 ***	Conscientiousness	0.192	0.071	6.75		0.000 ***	Wishful thinking	−0.461	−0.121	−5.69		0.000 ***
Social withdrawal	−0.143	−0.039	−3.85		0.000 ***	Problem avoidance	0.121	0.025	2.26		0.024 **	Extraversion	0.123	0.025	2.11		0.035 *
Conscientiousness	0.061	0.025	2.71		0.007 **	Self-criticism	−0.097	−0.027	−2.06		0.040 *						

N = Neuroticism; E = Extraversion; C = Conscientiousness; PS = Problem solving; SE = Self-criticism; EE = Emotional expression; WT = Wishful thinking; SS = Social support; CR = Cognitive restructuring; PA = Problem avoidance; SW = Social withdrawal; Models: Mental Health: (1) N; (2) N + E; (3) N + E + CR; (4) N + E + CR + WT; (5) N + E + CR + WT + SC; (6) N + E + CR + WT + SC + SW; (7) N + E + CR + WT + SC + SW + C; Vitality: (1) N; (2) N + E; (3) N + E + CR; (4) N + E + CR + WT; (5) N + E + CR + WT + C; (6) N + E + CR + WT + C + PA; (7) N + E + CR + WT + C + PA + SC; Emotional role functioning: (1) N; (2) N + C; (3) N + C + WT; (4) N + C + WT + SW; (5) N + C + WT + SW + EE; (6) N + C + WT + SW + EE + PA; (7) N + C + WT + SW + EE + E. *** *p* ≤ 0.001 ** *p* ≤ 0.01, * *p* ≤ 0.05.

## Data Availability

The data presented in this article are available on request from the corresponding author.
